# Experimental evaluation of femoral vein grafts and platelet-rich fibrin for reducing epidural fibrosis in a rat laminectomy model

**DOI:** 10.1038/s41598-026-54270-0

**Published:** 2026-05-30

**Authors:** Samah Fouad, Marwa Abass, Awad Rizk, Esam Mosbah, Mostafa Nabeeh, Walaa Awadin, Adel Zaghloul

**Affiliations:** 1 Medical Experimental Research Center (MERC), Faculty of Medicine, Mansoura University, Mansoura, 35516 Egypt; 2 Department of Surgery, Anesthesiology and Radiology, Faculty of Veterinary Medicine, Mansoura University, Mansoura, 35516 Egypt; 3 Department of Neurosurgery, Faculty of Medicine, Mansoura University, Mansoura, 35516 Egypt; 4 Department of Pathology, Faculty of Veterinary Medicine, Mansoura University, Mansoura, 35516 Egypt

**Keywords:** Laminectomy, Rats, PRF, Femoral vein graft, Diseases, Medical research

## Abstract

Epidural fibrosis (EF) refers to the non-physiological scar formation at the site where a surgical incision is made to access the spinal canal. This condition often results in functional disability and pain following spinal surgery and is a common and difficult-to-treat contributor to Failed Back Surgery Syndrome (FBSS). This study aimed to investigate the effectiveness of platelet-rich fibrin (PRF) clots, vein grafts, or a combination of both in reducing epidural fibrosis after laminectomy in rats. One hundred adult Sprague–Dawley male rats, weighing 300 ± 55 g, were equally and randomly assigned into five groups (n = 20): the control, laminectomy, PRF, vein graft, and PRF/vein graft groups. The control group consisted of normal, untouched rats that did not undergo anesthesia, surgical exposure, laminectomy, or sham operation. Lumbar laminectomy was performed at L3–L5 in the laminectomy, PRF, vein graft, and PRF/vein graft groups. On postoperative day 30, outcomes were assessed in two predefined independent subsets within each group. Ten rats per group were used for macroscopic epidural adhesion scoring after surgical-site reopening, whereas the remaining ten rats per group were used for histopathological, morphometric, and qRT-PCR analyses of IL-6 and TGF-β1 expression. Therefore, the effective analytical sample size was n = 10 animals per group for each endpoint category, not n = 20 per group. The primary endpoint was histological epidural fibrosis score at day 30; all other macroscopic, morphometric, inflammatory, and molecular outcomes were secondary endpoints. The EF area was significantly reduced in all treatment groups compared with the laminectomy group. Overall, the vein graft-containing groups showed the strongest reductions in adhesion, scar density, dura mater thickness, and IL-6/TGF-β1 expression. The combined PRF/vein graft treatment was generally more favorable than PRF alone but was often comparable to vein graft alone, indicating that a consistent superiority of the combined approach over vein graft alone was not demonstrated. Vein graft-containing treatments reduced histological and molecular markers of epidural fibrosis in this rat laminectomy model. The combined PRF/vein graft approach showed favorable anti-inflammatory and anti-fibrotic effects, but it was not consistently superior to the vein graft alone across all assessed endpoints. Therefore, the findings should be interpreted as preliminary preclinical evidence supporting further investigation of vein graft-based local barrier strategies rather than direct evidence of a superior combination therapy or a clinically ready approach.

## Introduction

Posterior lumbar decompression procedures, including laminectomy, hemilaminectomy, interlaminar fenestration, and minimally invasive or endoscopic approaches, are commonly used for the treatment of degenerative lumbosacral disorders. Although these techniques differ in the extent of bone and soft-tissue disruption, postoperative epidural fibrosis may still develop after surgical access to the spinal canal^1^. Postoperative back pain after lumbar decompression surgery can result from several causes, including foreign body reaction, recurrent disc herniation, pseudoarthrosis, spinal stenosis, root degeneration, spinal instability, epidural fibrosis, or incorrect surgical level^2^. Epidural fibrosis (EF) is a common and surgically challenging postoperative complication that may contribute to failed back surgery syndrome (FBSS). It can be defined as the extensive formation of fibrotic tissue in the epidural space, resulting in severe adhesion of nerve roots and the dura mater^3^.

Unfortunately, there have been no effective strategies for the treatment of EF, and the success rates of reoperations are low^1^. In the last decade, many studies have attempted to overcome this complication by using new methodologies, such as fat grafting, anti-inflammatory agents, Adcon-L, curcumin, collagen membranes, ozone treatment, and fibrinolytic agents. However, an effective treatment has not been discovered yet^4–9^.

Although various physical barriers and pharmacological agents have been investigated to prevent epidural fibrosis, many of these approaches have shown limited long-term efficacy and/or inconsistent outcomes across studies, partly due to variability in materials, dosing protocols, and experimental models^10^. Moreover, clinical translation remains limited, as most strategies have not progressed beyond preclinical settings, and none has become a standardized preventive modality in routine spinal surgery. These limitations highlight the ongoing need for alternative biologically based approaches that can be evaluated in well-controlled preclinical models^11–13^.

The previous research^14–16^ suggested that human arteries were successfully used as a graft to regenerate a sciatic nerve injury in a dog. The axon would grow through the vessel wall with minimal scar tissue. Although these studies were conducted in the context of peripheral nerve regeneration and are not directly equivalent to epidural fibrosis after laminectomy, they suggest that autogenous vascular tissue may act as a biocompatible scaffold or barrier. Therefore, in the present study, the femoral vein graft was investigated as an experimental local barrier placed over the exposed dura rather than as a proven mechanistic anti-fibrotic therapy^17,18^.

Autogenous vein grafts are commonly preferred because they eliminate the need for a donor and reduce the risk of complications related to the body’s immune response^15^. In this setting, a vein graft may function as a biocompatible physical barrier and biological scaffold that can modulate local healing, thereby potentially limiting fibroblast infiltration and adhesion formation around the dura^19^.

From a biological and technical perspective, combining an autogenous vein graft with autologous PRF represents a feasible experimental approach: the vein can provide a biocompatible barrier/scaffold, while PRF may modulate early postoperative inflammation and tissue repair; importantly, PRF has also been investigated experimentally for reducing epidural fibrosis after laminectomy^20,21^.

Vascular grafting is frequently utilized in a variety of clinical and surgical treatments, including carotid revascularization, coronary artery and cardiopulmonary bypass, free-flap transplantation and replantation, peripheral nerve repair, and revascularization of abnormalities in the extremities. There are two types of vascular grafts: arterial and venous. Arterial sources for grafts include the tail artery (middle caudal artery), the aorta, the left and right carotid arteries, the femoral arteries, and the saphenous arteries. Venous graft sources included the left and right jugular veins, femoral veins, saphenous veins, and superficial epigastric veins^22^.

Recently, new substances, such as platelet-rich fibrin (PRF), have been developed for surgical nerve injury repair. PRF is a platelet and an immune concentrate containing all the beneficial components found in a blood sample that promote immunity and healing^23^. PRF can reduce the density of chronic inflammatory cells after laminectomy in rats. Moreover, it can enhance tissue healing, angiogenesis, and osteoblast proliferation by releasing cytokines and growth factors from platelets^24^.

Platelet-derived growth factor (PDGF), transforming growth factor-beta1 (TGF-β1), epidermal growth factor, and vascular endothelial growth factor (VEGF) are among the growth factors (GFs) that PRF can release via platelet activation for up to 28 days following application.^25^

To the best of the author’s knowledge, there have been no previous studies examining the use of femoral vein grafts for alleviating EF post-laminectomy in either humans or experimental animals. Thus, the purpose of this experimental investigation was to assess whether a femoral vein graft, alone or combined with PRF, could reduce histological, morphometric, and molecular features of epidural fibrosis after laminectomy in rats. The study was designed as a preclinical proof-of-concept experiment and was not intended to establish clinical efficacy.

## Materials and methods

### Animals and groups

This study utilized a cohort of 100 adult male Sprague Dawley rats weighing 300 ± 55 g obtained from the Medical Experimental Research Center of Mansoura University (MERC). The experimental protocol of this work was approved by the Mansoura University Animal Care and Use Committee with registration number MU-ACUC (VM.PhD.24.11.50). All animal procedures were performed in accordance with the relevant guidelines and regulations. This experimental study complied with the ARRIVE guidelines.

Sample size estimation was performed using G*Power for the primary between-group comparison related to the histological epidural fibrosis score. The calculation was based on one-way ANOVA, fixed effects, omnibus test, five groups, α = 0.05, power = 80%, and a large expected effect size (f = 0.50). This calculation indicated a required total sample size of approximately 53 animals, corresponding to about 11 animals per group for a single endpoint analysis. In the present study, the animals were prospectively divided into two independent endpoint-specific subsets to avoid tissue disruption from repeated surgical-site reopening. Therefore, although the total number of animals used in the whole experiment was 100, the effective analytical sample size was 50 animals per endpoint, corresponding to n = 10 animals per group for macroscopic assessment and n = 10 animals per group for histological, morphometric, and molecular analyses. Thus, the total animal number should not be interpreted as the analytical sample size for any single endpoint. The primary endpoint was the histological epidural fibrosis score assessed on postoperative day 30 in the predefined histology/molecular subset. Secondary endpoints included macroscopic epidural adhesion score, inflammation grade, scar density score, dura mater thickness, and mRNA expression levels of IL-6 and TGF-β1.

The rats were randomly assigned to five groups, with 20 rats in each group. Randomization was performed using a computer-generated random number sequence by an investigator not involved in surgery or outcome assessment. Group allocation was concealed in sequentially numbered, sealed opaque envelopes that were opened immediately before intervention preparation. Because the surgeon had to prepare and apply the assigned treatment, surgical blinding to group allocation was not feasible. However, all macroscopic, histological, morphometric, and molecular outcome assessments were performed by investigators blinded to group allocation. Each group was prospectively subdivided into two independent assessment sets (n = 10 each): 10 rats were allocated for macroscopic epidural adhesion scoring, and 10 rats were reserved for histopathological, morphometric, and molecular analyses. This separation was planned to avoid tissue disruption from surgical site reopening, which could interfere with downstream histological and gene expression evaluations. The study included a normal, untouched control group, which did not undergo anesthesia, skin incision, paraspinal muscle dissection, laminectomy, or sham surgical exposure. This group was used only to provide baseline normal histological and molecular values. Because no sham-operated group was included, the normal control group could not be used to isolate the effects of anesthesia, surgical exposure, muscle dissection, bleeding, or wound healing from the effects of laminectomy. The laminectomy group underwent laminectomy without any anti-fibrotic treatment and was therefore used as the surgical comparator group. The PRF, vein graft, and PRF/vein graft groups underwent laminectomy followed by their assigned treatments. Before surgery, autologous PRF clots and/or autologous femoral vein grafts were prepared only from the same rats assigned to the relevant treatment groups.

### Preparation of the autogenous PRF

**Fig. 1:**
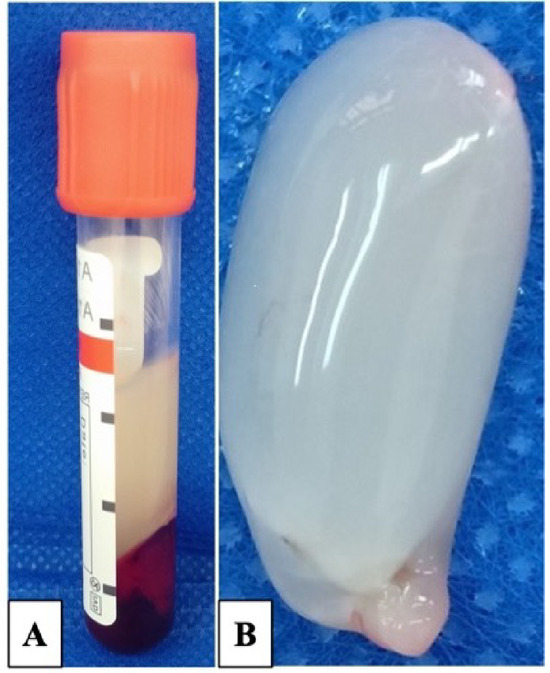
Autologous PRF clot preparation. (**A**): Blood sample after centrifugation, the PRF clot adhered to RBCs. (**B**): Autologous PRF clot.

A volume of 3 mL of blood was collected from the caudal tail vein of the rats in either the PRF or PRF/vein graft groups. The blood was extracted into a glass centrifuge tube (Shanghai Goldenwell Medical Technology Co, Ltd., China) without the use of an anticoagulant^26^. Subsequently, the tubes were promptly subjected to centrifugation at a speed of 3000 revolutions per minute (rpm) for 10 min (Hettich EBA 8S centrifuge, D-78532 Tuttlingen, Germany) (RCF = 402 × g) with a 450-rotor angulation and a radius of 40 mm. Three centrifugation strata were produced: the acellular plasma at the top, the red corpuscles at the bottom, and a fibrin clot (PRF) in the center of the tube. The large fibrin networks caught a considerable number of platelets. The center fraction was gathered after the top layer, which looked like straw, was removed. After that, the serum from the clot was extracted to create a strong, self-derived fibrin membrane (Fig. 1).

### Femoral vein grafting

**Fig. 2:**
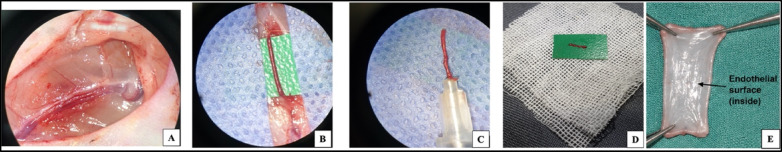
Preparation of the femoral vein graft. (**A**) Three vital structures are visible after the surgical incision of the right hindlimbs of the rat’s femoral vein, artery, and nerve. All structures are enclosed within a connective tissue sheath. (**B**) The femoral vein was dissected and isolated on a sterile surgical pad, then the distal and proximal ends of the femoral vein were ligated. (**C**): A 5 mm segment of the femoral vein was caught by a needle of the insulin syringe and cut. (**D**) The femoral vein graft was kept on the sterile wet gauze. (**E**): The femoral vein graft was longitudinally opened to expose the inner endothelial surface before placement over the dura mater.

Autologous femoral vein grafts were harvested from the same rats assigned to the vein graft and PRF/vein graft groups. The right hind limb was surgically prepared according to^27^. Microsurgical procedures were performed using a surgical microscope (LEICA M50; Model MEB113; Singapore) to dissect and ligate the femoral vein. Two 6/0 polyglactin 910 sutures (Vicryl; Ethicon; NJ, USA) were placed around the vein. A femoral vein segment measuring approximately 5 mm was then harvested. A longitudinal incision was made along the femoral vein segment to expose the inner endothelial surface. After longitudinal opening, the autologous femoral vein graft was placed over the exposed dura mater with the endothelial surface facing the dura and the adventitial surface facing dorsally (Fig. 2).

### Surgical operation (laminectomy)

Rats were administered an intramuscular antibiotic injection (Ampicillin, 15 mg/200 g BW). In addition, Ophthalmic Ointment (Terramycin; Pfizer; Egypt) was applied to the eyes to prevent drying, and the rats were placed on a heating pad to maintain a body temperature of 37 °C. Immediately prior to surgery, the surgical site (the back of the rat) was aseptically prepared.

**Fig. 3:**
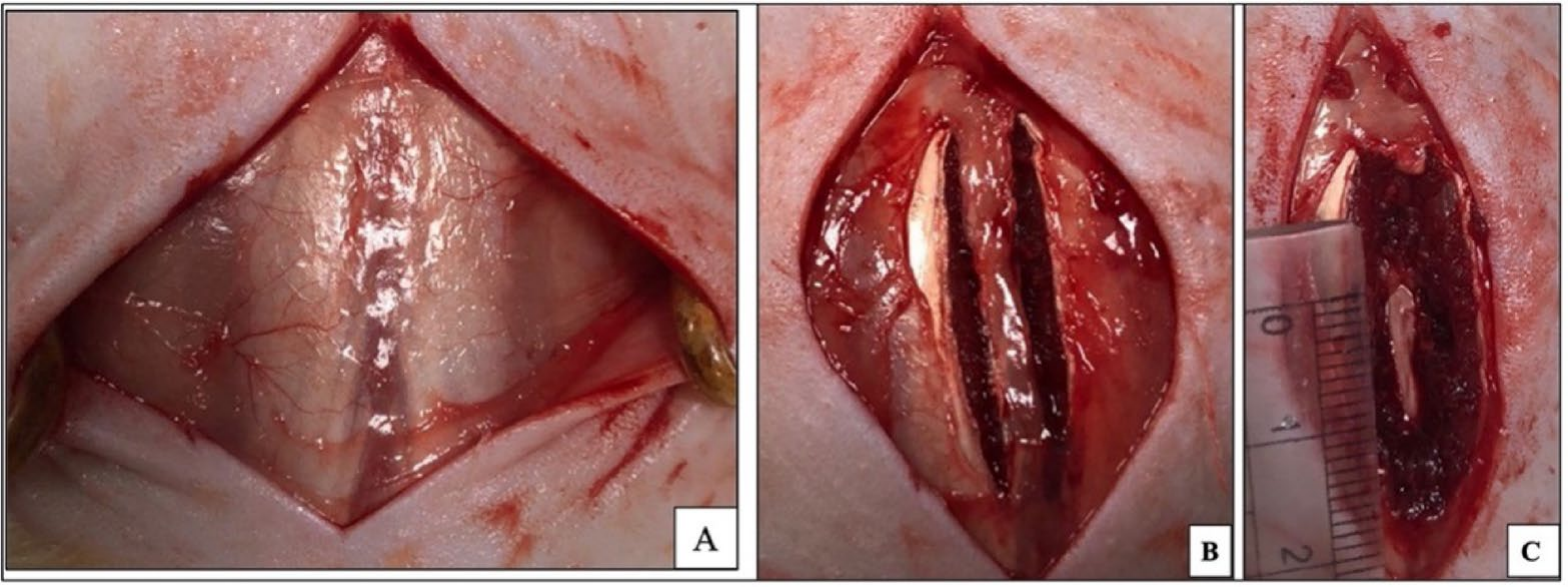
Surgical lumbar laminectomy (L3-L4) in the rat. (**A**): A longitudinal midline skin incision was performed over the dorsal spinous processes of the L3 up to L5. (**B**): The lumbosacral fascia was incised longitudinally, and the paraspinal muscles were dissected to expose the L3–L5 laminae. (**C**): A careful and complete laminectomy was conducted, followed by the excision of the ligamentum flavum and epidural adipose tissue.

Rats were generally anesthetized using an intraperitoneal injection of a mixture of 5 mg/kg Xylazine hydrochloride (Xylajet 20 mg/ml, ADWIA, Egypt) and 75 mg/kg ketamine hydrochloride (Ketamax 50 mg/ml, Troikaa Pharmaceuticals Ltd, Gujarat, India). The rats were placed in a prone position. All surgical procedures were performed by M.A. under a surgical microscope (LEICA M50; Model MEB113; Singapore). Following the sterile preparation of their lower backs, a longitudinal midline skin incision was performed over the dorsal spinous processes of the L3 and L5. The lumbosacral fascia was then incised longitudinally, followed by the dissection of paraspinal muscles on both sides in a subperiosteal manner to expose the L3–L5 laminae. A complete laminectomy was conducted at the L3 vertebra level, followed by flavectomy (the excision of the ligamentum flavum) and the epidural adipose tissue from the surgical area. Carefully use a rongeur to remove the spinous process and vertebral plate to avoid damaging the dura mater and nerve roots (Fig. 3). The dura mater was fully exposed, left intact, and kept clean. Clean cotton pads were used to achieve hemostasis. The rats were then treated according to their predefined group allocation. After treatment, the surgical wounds were sutured in anatomical layers using multiple simple interrupted sutures of 5/0 polyglactin 910 (Vicryl; Ethicon; NJ, USA).

The surgical staple was used to close the skin incision. After the surgery, the topical antibiotic Fucidin (30 g cream 2%, MINAPHARM, Egypt) was applied to the incision site. Each rat was singly housed until fully recovered from anesthesia; then, the rats were returned to their cages the next day and supplemented with softened food and water.

On the 30^th^ day post-operation, the rats were euthanized by intraperitoneal administration of thiopental sodium (100 mg/kg; 500 g thiopental, EIPCO, Egypt). Following euthanasia, perfusion was carried out using a 4% paraformaldehyde solution. From each group, ten rats were allocated to the macroscopic assessment subset, in which the surgical site was carefully reopened for epidural adhesion scoring. The remaining ten rats from each group were allocated to the histology/molecular subset and were not subjected to surgical-site reopening before tissue collection; these animals were used for histopathological, morphometric, and qRT-PCR analyses.

### Assessment and grading of epidural fibrosis (EF)

On postoperative day 30, endpoint assessments were performed according to the predefined subset allocation. Macroscopic epidural adhesion scoring was performed only in the macroscopic subset (n = 10/group), whereas histological, morphometric, and molecular assessments were performed only in the histology/molecular subset (n = 10/group). The predefined primary endpoint was the histological epidural fibrosis score. Secondary endpoints included macroscopic epidural adhesion score, inflammation grade, scar density score, dura mater thickness, and IL-6/TGF-β1 mRNA expression. All macroscopic and histological scoring was performed independently by two experienced assessors blinded to group allocation.

#### Macroscopic evaluation of epidural adhesion score (EAS)

After carefully reopening the surgical sites for macroscopic examination, the adhesion of the epidural scar was assessed using the Rydell classification^27^. The following grades are included in this classification scheme: Epidural scar tissue adhering to the dura mater is indicated by a grade of zero; adhering to the dura mater but being easily dissected is indicated by a grade of one; adhering to the dura mater but being difficult to separate without causing damage to the dura mater is indicated by a grade of two; and adhering to the dura mater but being unable to be dissected is indicated by a grade of three.

#### Histopathological analysis by (H&E)

Histopathological evaluation was performed only in the predefined histology/molecular subset (n = 10/group), which was not subjected to surgical-site reopening before tissue collection. The en-bloc vertebral columns between L2 and L6 were removed and placed into a 10% neutral-buffered formaldehyde solution for five days. The specimens were subsequently decalcified in 10% EDTA (Sigma Aldrich, St Louis, MO, USA) for three weeks^28^ and then embedded in paraffin. Three consecutive sections were obtained from the distal, middle, and proximal parts of the region and then placed in sampling cassettes. Additionally, 5 μm axial sections of the laminectomy site were stained with H&E (Shandon Harris Hematoxylin–Eosin Y; Fisher Scientific, Leicestershire, United Kingdom) and Masson trichrome^29^.

#### Quantitative morphometric analysis by (MT)

The Masson’s trichrome-stained sections were subjected to quantitative morphometric analysis, including the epidural adhesion and fibrosis, inflammation grade, scar density grade, and dura mater thickness grade, using image analysis software (Image J, 1.46a, NIH, USA). Morphometric outcomes (e.g., epidural thickness and dura mater thickness) were quantified using ImageJ measurements in standardized fields and magnifications.

The extent of inflammation was graded in H&E sections as follows: 0 = absent inflammation, 1 = mild cellular infiltrate of neutrophils, possibly mixed with a few lymphocytes and mild tissue edema, 2 = moderate mixed cellular infiltrate with noticeable tissue edema may be noticed, and 3 = severe cellular infiltrate with marked edema and altered normal tissue pattern^30^.

Additionally, the scar density was assessed based on myofibroblast differentiation and categorized into grades: Grade 0 for no scar formation, Grade 1 for differentiated myofibroblasts less than 10%, Grade 2 for differentiated myofibroblasts 11–50%, Grade 3 for differentiated myofibroblasts 51–75%, and Grade 4 for differentiated myofibroblasts greater than 76%^31^.

Moreover, the dura mater thickness was assessed at three different levels using a magnification of 400X, and the presence of arachnoid involvement was recorded^32^.

Under the light microscope, the epidural adhesion, fibrosis, and other histological changes were identified^30^. Grade 0 indicates that there is no scar tissue covering the dura mater, while Grade 1 indicates the presence of a thin fibrous band between the dura mater and scar tissue. Grade 2 refers to the presence of scar tissue covering less than two-thirds of the laminectomy defect. Grade 3 indicates that scar tissue covers more than two-thirds of the laminectomy defect and extends to the arachnoids and nerve roots.

### qRT-PCR analysis of IL-6 and TGF-β1 relative mRNA expression

qRT-PCR analysis was performed using scar tissue collected only from the predefined histology/molecular subset (n = 10/group), not from animals used for macroscopic reopening and adhesion scoring. The mRNA levels of IL-6 and TGF-β1 were assessed according to^33^. The scar tissues from the sites where the laminectomy was performed were collected. Total RNA extraction was done utilizing Trizol reagent, and 2 μg of RNA was transcribed into cDNA utilizing a Promega reverse transcriptase kit. The qRT-PCR was performed in triplicate using the Bio-Rad MYIQ2 system, following the methodology described in a previous study. The primer sequences for the investigated genes are provided in Table 1. The amplification of GAPDH was utilized as an internal control. Relative mRNA expression was calculated using the 2^-ΔΔCt method after normalization to GAPDH and expressed as fold change relative to the control group.

**Table 1:** The primer sequences for the studied genes (IL-6 and TGF-β1).

Gene	Sequence	Product Size
GAPDH	Forward: CTCTGCTCCTCCCTGTTCTA Reverse: AGTTGAGGTCAATGAAGGGG	187
IL-6	Forward: GAAGTTAGAGTCACAGAAGGAGTG Reverse: GTTTGCCGAGTAGACCTCATAG	105
TGF-β1	Forward: TGCTAATGGTGGACCGCAA Reverse: CACTGCTTCCCGAATGCTGA	101

### Statistical analysis

The results were analyzed using SPSS software version 22.0 (IBM Corp., USA). Data distribution was assessed using the Shapiro–Wilk test, supported by visual inspection of histograms and Q–Q plots. Homogeneity of variance was evaluated before applying parametric tests.

Continuous quantitative variables with normal distribution were expressed as mean ± standard error of the mean (SE) and compared among groups using one-way analysis of variance (ANOVA), followed by Tukey’s post-hoc test for pairwise comparisons. For non-normally distributed quantitative variables, comparisons were performed using the Kruskal–Wallis test followed by Dunn’s post-hoc test with adjustment for multiple comparisons.

Ordinal semi-quantitative scores, including inflammatory scores, scar density scores, epidural fibrosis scores, and epidural scar adhesion scores, were analyzed using the Kruskal–Wallis test followed by Dunn’s adjusted pairwise comparisons. Grade distributions were also presented as frequencies and percentages to clarify the distributional pattern within each group. Because several contingency-table cells had small expected counts, distributional comparisons of grade frequencies were performed consistently using Fisher–Freeman–Halton exact testing rather than alternating between Pearson chi-square and likelihood-ratio tests. These exact tests were used only to support the descriptive interpretation of grade distributions, whereas the main inferential analysis of ordinal severity scores was based on Kruskal–Wallis testing followed by Dunn’s adjusted pairwise comparisons. Effect sizes were reported as epsilon-squared (ε^2^) for Kruskal–Wallis outcomes and Cramer’s V for grade-distribution analyses where applicable. For continuous outcomes, 95% confidence intervals were reported. For continuous outcomes analyzed by one-way ANOVA, effect size was expressed as eta-squared (η^2^), calculated as the ratio of between-group sum of squares to total sum of squares.

For omnibus tests, overall *P* values were reported first, followed by adjusted pairwise *P* values where applicable. Exact *P* values were reported whenever possible, and adjusted *P* ≤ 0.05 was considered statistically significant. Different superscript lowercase letters indicate statistically significant differences between groups at adjusted *P* ≤ 0.05.

Inter-observer agreement for semi-quantitative scoring was assessed using Cohen’s kappa coefficient. Sample size estimation was performed a priori using G*Power for the primary between-group comparison related to the predefined primary endpoint, namely the histological epidural fibrosis score. Because macroscopic and histology/molecular outcomes were assessed in predefined independent subsets, all endpoint-specific analyses were performed using n = 10 animals per group. The total number of animals in the study was 100, but the effective analytical sample size was 50 animals for each endpoint category. Therefore, statistical power and interpretation were based on the endpoint-specific sample size rather than on the overall animal number. Secondary endpoints were analyzed as supportive outcomes. Post-hoc multiplicity adjustment was applied within each endpoint for pairwise comparisons; however, no formal multiplicity adjustment was applied across secondary endpoints, which were interpreted as supportive rather than independent primary efficacy outcomes.

## Results

### Macroscopic assessment of epidural adhesion score (EAS)

**Fig. 4:**
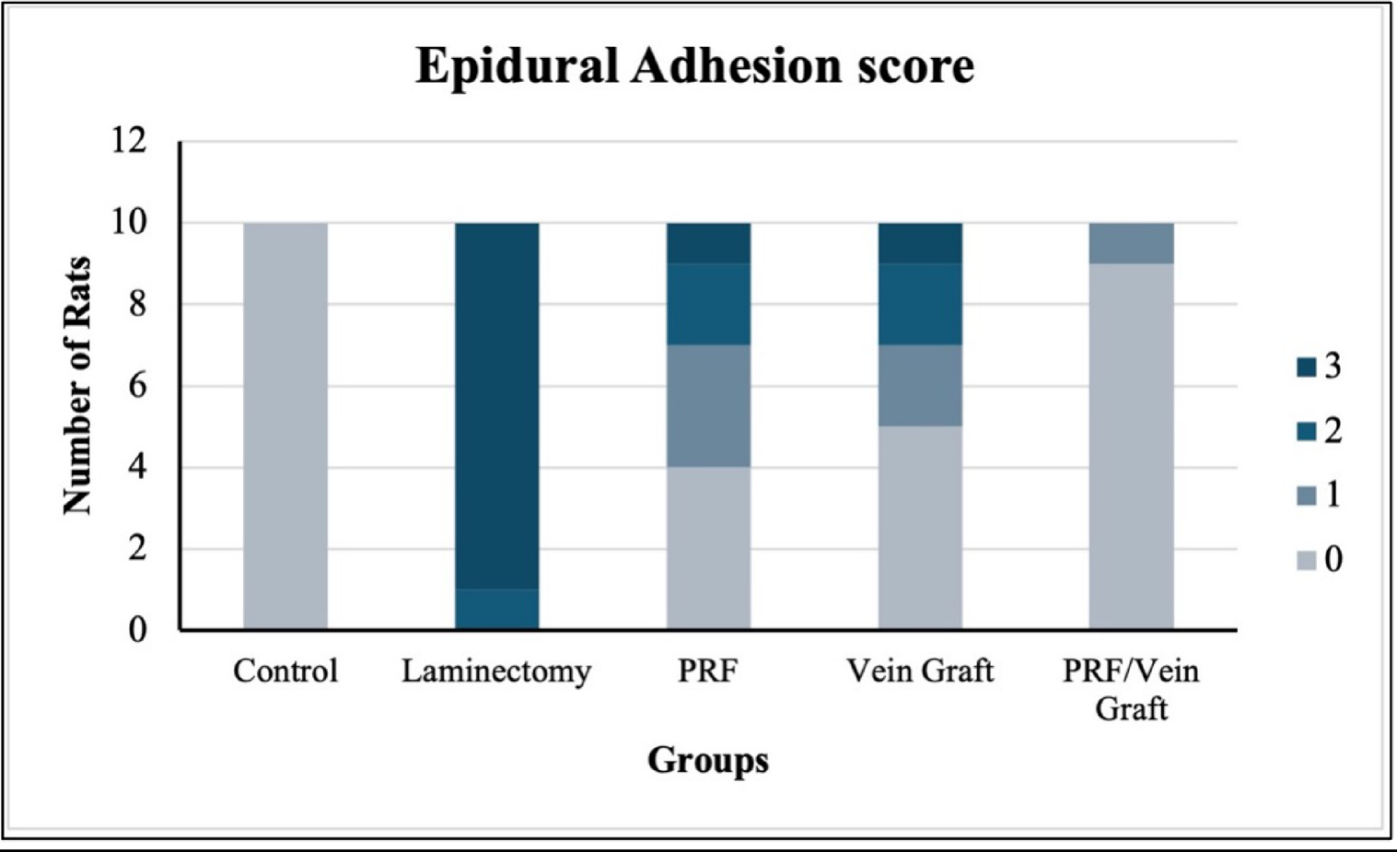
Epidural adhesion score (EAS) between groups based on Rydell’s classification. Data are presented as ordinal scores with grade distributions. Overall differences were analyzed using Kruskal–Wallis testing followed by Dunn’s adjusted post-hoc comparisons. Grade distributions were additionally summarized as frequencies and percentages, with exact testing used for distributional comparisons where applicable. Different lowercase letters indicate statistically significant differences at adjusted *P* ≤ 0.05. Data are from the macroscopic assessment subset (n = 10/group).

Macroscopic epidural adhesion scoring was performed in the predefined macroscopic subset (n = 10/group). The epidural adhesion score differed significantly among the groups using the Kruskal–Wallis test (H = 39.89, overall *P* < 0.0001, ε^2^ = 0.80; Fig. 4), indicating a large between-group effect.

Grade distribution analysis also demonstrated a strong treatment-related shift in adhesion severity (Cramer’s V = 0.65). The laminectomy group showed the most severe adhesion pattern, with grade 3 adhesion observed in 90% of animals. In contrast, both the vein graft and PRF/vein graft groups showed grade 0 adhesion in 90% of animals, while the PRF group showed a partial reduction in adhesion severity, with 20% grade 0, 50% grade 1, 20% grade 2, and 10% grade 3. Pairwise exact distributional comparisons showed that the laminectomy group differed significantly from the PRF group (*P* = 0.001), vein graft group (*P* < 0.0001), and PRF/vein graft group (*P* < 0.0001). The PRF/vein graft group did not differ from the vein graft group (*P* = 1.000), but both showed lower adhesion severity than the PRF group (*P* = 0.014). These findings indicate that the biological effect was not limited to statistical significance, but was reflected by a clear shift from severe grade 3 adhesion toward absent or minimal adhesion.

### Histopathological analysis (H&E)

**Fig. 5:**
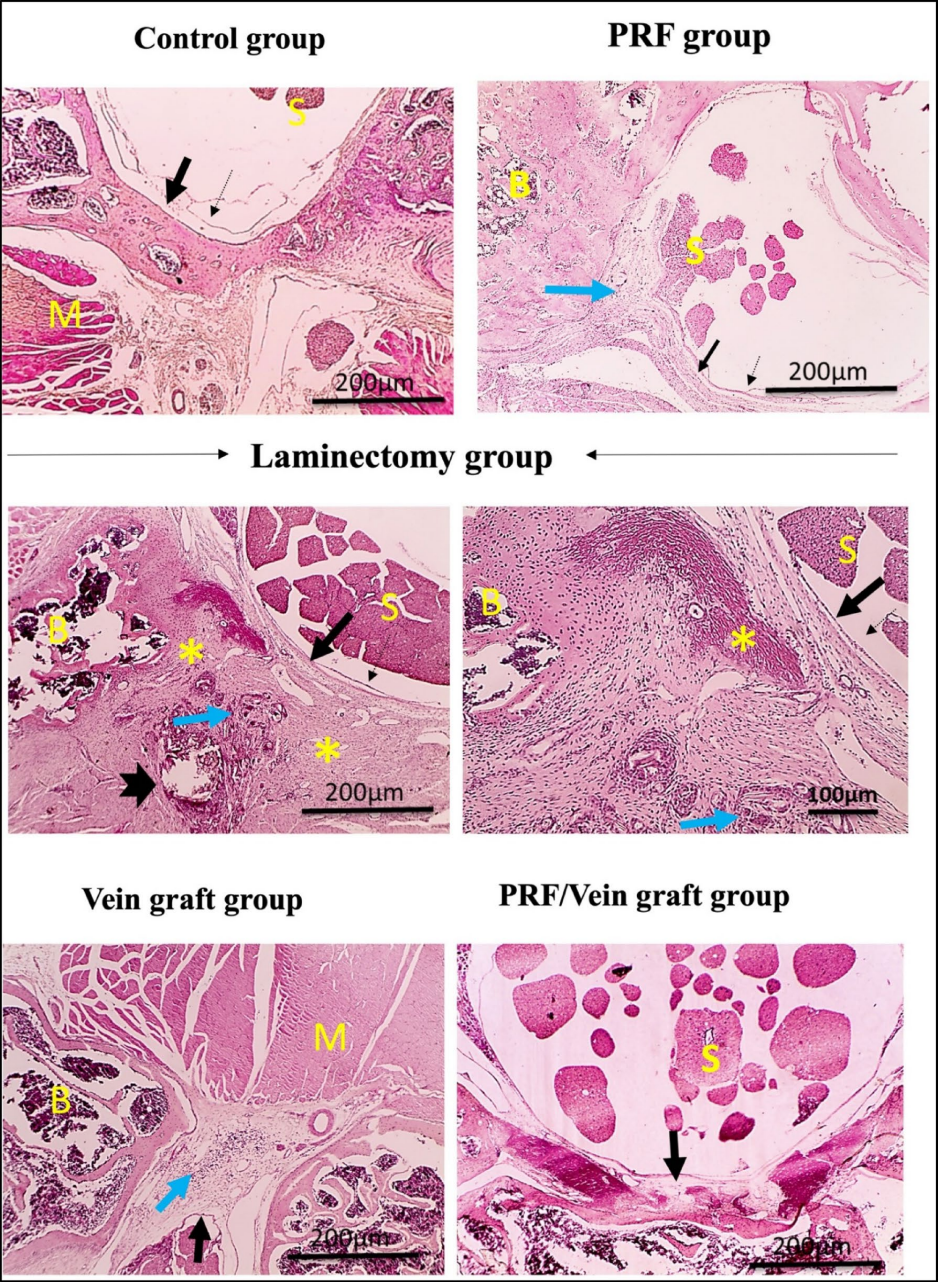
Microscopic pictures showing normal structures of arachnoid (dashed black arrow); Dura mater (black arrow); B: Bone; S: spinal cord and spinal roots, including parts of the cauda equina; M: muscle in the control normal group. Epidural thickness increased in the laminectomy group, besides excessive deposition of scar tissue (*) with or without adherence to Dura mater and granuloma formation (blue arrow) and dystrophic calcification (thick black arrow). Inflammation markedly decreased (blue arrow) in the vein graft group, moderately decreased (blue arrow) in the PRF group, and was absent in the PRF/vein graft group. H&E, X: 40 bar 200 m and X: 100 bar 100 μm.

**Fig. 6:**
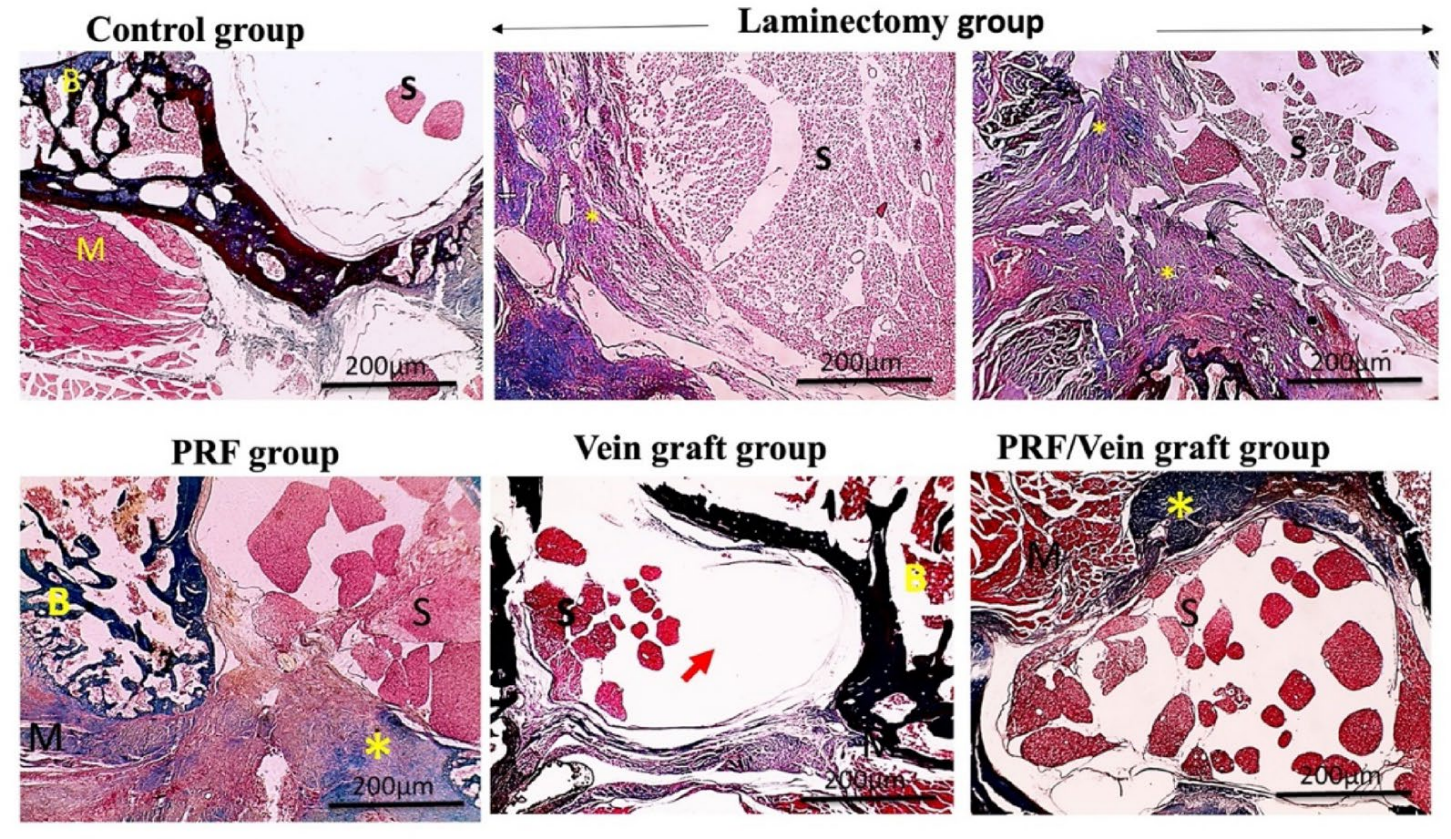
The MT stain revealed an increased epidural thickness in the laminectomy group, as well as excessive deposition of scar tissue (*) with (thin blue arrows) or without adherence to the dura mater. Epidural thickness and amount of scar tissue (*) decreased in all treated groups compared to the laminectomy group. There was no adherence to the dura mater in the vein graft group or the PRF/vein graft group. Focal adherence to the dura mater (thin blue arrows) is seen in the PRF group. Arachnoid (dashed black arrows); Dura mater (thin black arrows); Femoral vein graft (red arrows); B: Bone; S: spinal cord and spinal roots, including parts of the cauda equina; M: Muscle. MT, X: 40 bar 200 μm.

The control group exhibited the normal structures of the arachnoid, dura mater, bone, spinal cord, and muscle (Fig. 6). In the laminectomy group, increased epidural thickness, besides excessive deposition of scar tissue with or without adherence to dura mater, granuloma formation, and dystrophic calcification were demonstrated in (Fig. 5). In comparison to the laminectomy group, markedly decreased inflammation is observed in the vein graft group, moderately decreased inflammation is observed in the PRF group, and absent inflammation is observed in the PRF/vein graft group. Moreover, the inflammation scores significantly decreased in all the treated groups (Fig. 5).

### Quantitative morphometric analysis by (MT)

**Fig. 7:**
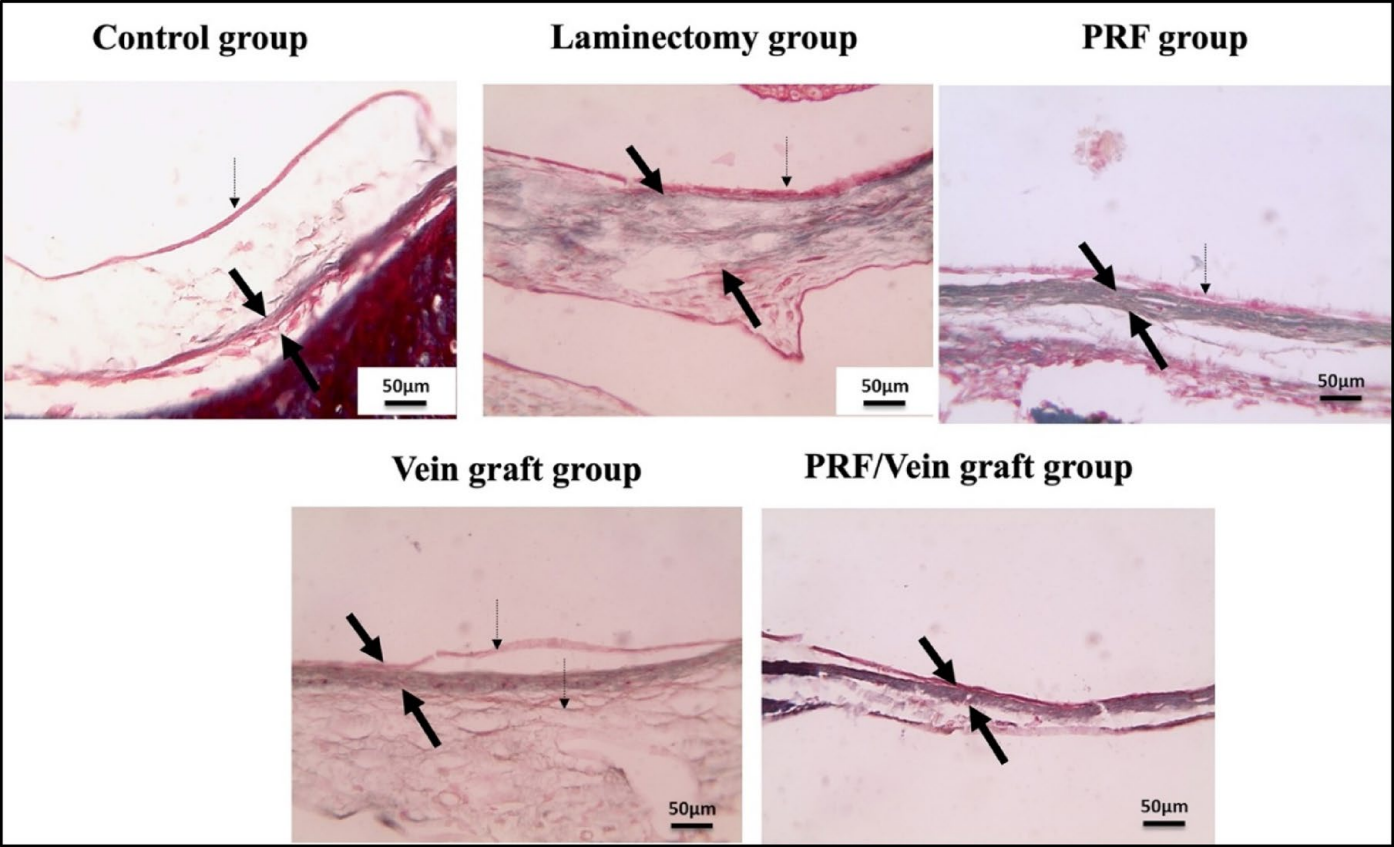
The MT satin showed a decreased epidural thickness in all the treated groups compared to the laminectomy group. Arachnoid (dashed black arrows); Dura mater (thin black arrows); X: 400 bar 50 μm.

Histological and morphometric analyses were performed in the predefined histology/molecular subset (n = 10/group). Decreased epidural thickness and amount of scar tissue were seen in all the treated groups compared to the laminectomy group. No adherence to dura mater was shown in the vein graft group and PRF/vein graft group, while focal adherence to dura mater was detected in the PRF group (Fig. 6). Scores of scar tissue density showed a significant decrease in the vein graft group and PRF/vein graft group compared to the laminectomy group (Fig. 6). In all the treated groups, epidural thickness decreased compared to the laminectomy group (Fig. 7).

The inflammatory score differed significantly among the groups using the Kruskal–Wallis test (H = 38.67, overall *P* < 0.0001, ε^2^ = 0.77; 8A), indicating a large treatment effect. Grade distribution analysis also supported a strong shift toward lower inflammatory grades in the treated groups (Cramer’s V = 0.70). The laminectomy group showed the highest inflammatory severity, with 30% of animals showing grade 2 inflammation and 70% showing grade 3 inflammation. In contrast, the PRF/vein graft group showed the mildest inflammatory pattern, with 40% grade 0 and 60% grade 1 inflammation, and no animals showing grade 2 or grade 3 inflammation. The PRF group also showed a marked reduction in inflammation, with most animals classified as grade 0 or grade 1. Pairwise exact comparisons showed significantly lower inflammatory grades in the PRF group (*P* < 0.0001), vein graft group (*P* = 0.001), and PRF/vein graft group (*P* < 0.0001) compared with the laminectomy group. The PRF/vein graft group also showed lower inflammatory severity than the vein graft group (*P* = 0.010), while its difference from the PRF group was not statistically significant (*P* = 0.680). These results show both statistical and distributional evidence of reduced postoperative inflammation after treatment.

**Fig. 8:**
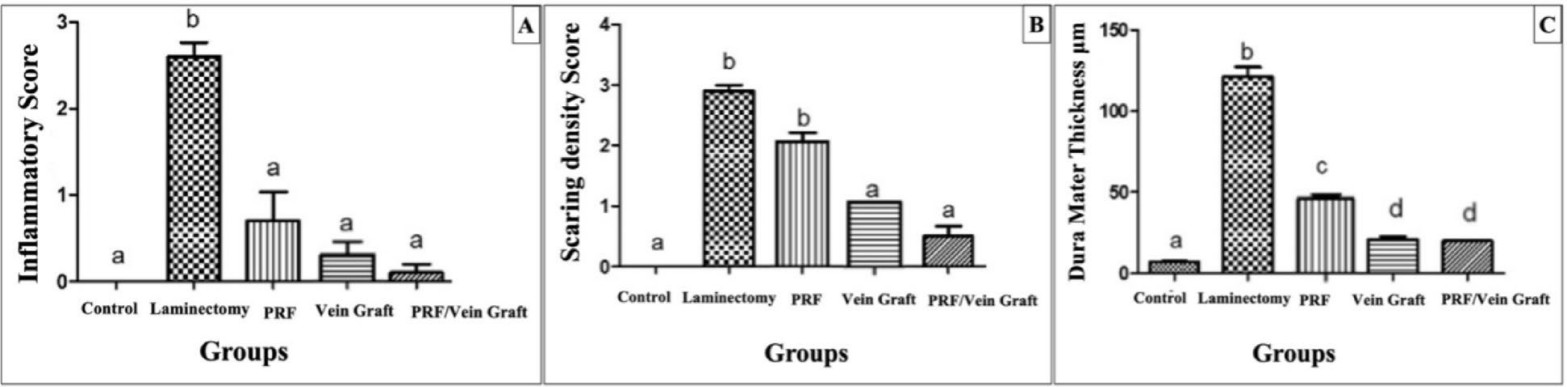
Inflammation scores (**A**) were analyzed using the Kruskal–Wallis test followed by Dunn’s adjusted post-hoc comparisons and showed a significant decrease in the treatment groups compared with the laminectomy group. Scar tissue density scores (**B**) were analyzed using the Kruskal–Wallis test followed by Dunn’s adjusted post-hoc comparisons and revealed a significant decrease in the vein graft and PRF/vein graft groups compared with the laminectomy group. Dura mater thickness (**C**) was analyzed using one-way ANOVA followed by Tukey’s adjusted post-hoc test and showed a significant reduction in all treated groups compared with the laminectomy group. Different lowercase superscript letters indicate statistically significant differences between groups based on adjusted post-hoc comparisons at *P* ≤ 0.05. Data are from the histology/molecular subset (n = 10/group).

The scar tissue density score differed significantly among the groups using the Kruskal–Wallis test (H = 35.70, overall *P* < 0.0001, ε^2^ = 0.70; Fig. 8B), indicating a treatment-related reduction in fibrotic tissue organization. Dunn’s adjusted post-hoc comparisons showed that scar density scores were significantly lower in the vein graft group and PRF/vein graft group compared with the laminectomy group (adjusted *P* ≤ 0.05). The PRF group also showed a reduction compared with the laminectomy group, although this reduction was less pronounced than that observed in the vein graft-containing groups. The greatest reduction was observed in the vein graft and PRF/vein graft groups, supporting that the treatment effect involved not only a decrease in scar presence but also a meaningful reduction in scar severity.

One-way ANOVA showed a significant overall difference among groups in dura mater thickness (F(4,45) = 41.75, *P* < 0.0001, η^2^ = 0.79). Dura mater thickness was markedly increased in the laminectomy group (141.3 ± 5.12 μm; 95% CI 129.72–152.88 μm) compared with the control group (19.3 ± 4.21 μm; 95% CI 9.78–28.82 μm). All treated groups showed a clear reduction in dura mater thickness compared with the laminectomy group, including the PRF group (56.6 ± 10.04 μm; 95% CI 33.89–79.31 μm), vein graft group (31.7 ± 7.59 μm; 95% CI 14.53–48.87 μm), and PRF/vein graft group (33.0 ± 9.45 μm; 95% CI 11.62–54.38 μm). Tukey’s post-hoc comparisons confirmed significant reductions versus the laminectomy group for PRF, vein graft, and PRF/vein graft treatments (all *P* < 0.001). The mean differences from the laminectomy group were 82.0 μm for PRF, 97.0 μm for vein graft, and 97.67 μm for PRF/vein graft, indicating a substantial magnitude of reduction rather than a minor statistically significant change Fig. 8C).

**Fig. 9:**
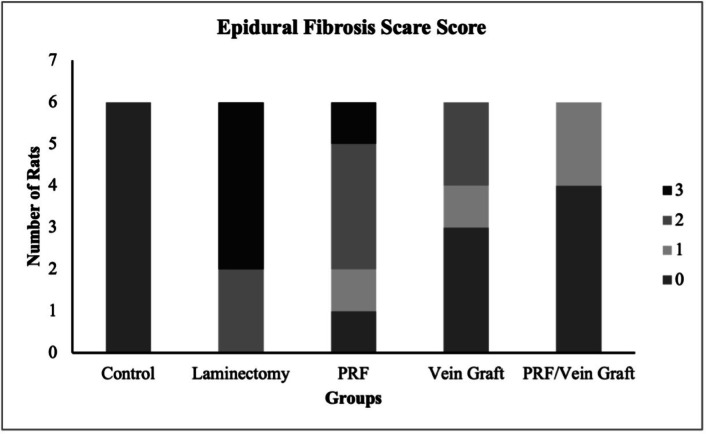
Epidural fibrosis score (EFS) between groups. Data are from the histology/molecular subset (n = 10/group).

The epidural fibrosis score differed significantly among the groups using the Kruskal–Wallis test (H = 33.52, overall *P* < 0.0001, ε^2^ = 0.66; Fig. 9), indicating a treatment-related effect on fibrosis severity. Grade distribution analysis also showed a marked treatment-related shift toward lower fibrosis grades (Cramer’s V = 0.62). Dunn’s adjusted post-hoc comparisons showed that the laminectomy group had significantly higher fibrosis scores than the treated groups (adjusted *P* ≤ 0.05). The laminectomy group showed the highest fibrosis severity, with grade 3 fibrosis observed in seven animals, whereas the PRF/vein graft group showed the lowest fibrosis scores among the surgical groups, with most animals showing grade 0 fibrosis. The PRF/vein graft group was not significantly different from the control group, suggesting near-normal histological appearance in this endpoint. The vein graft group showed lower fibrosis scores than the laminectomy group but remained significantly different from the control group, while the PRF group showed higher fibrosis scores than the vein graft and PRF/vein graft groups, indicating a smaller anti-fibrotic effect compared with vein graft-containing treatments. Inter-observer agreement for histological epidural fibrosis grading was substantial (Cohen’s κ = 0.63).

Direct comparison among the treatment groups showed that the PRF/vein graft group produced outcomes that were generally better than PRF alone and comparable to vein graft alone. For the macroscopic epidural adhesion score, the PRF/vein graft group did not differ significantly from the vein graft group, whereas both vein graft-containing groups showed lower adhesion severity than the PRF group. For the inflammatory score, the PRF/vein graft group showed lower inflammatory severity than the vein graft group, while it was not significantly different from the PRF group. For scar density, epidural fibrosis score, dura mater thickness, and IL-6/TGF-β1 expression, the combined treatment showed marked reductions compared with the laminectomy group and followed a pattern close to the vein graft group. Taken together, these direct treatment-group comparisons indicate that the vein graft-containing groups produced the strongest overall anti-fibrotic pattern. Although the combined PRF/vein graft treatment showed favorable results and was generally better than PRF alone, it was not consistently superior to vein graft alone across the assessed endpoints. Therefore, the data support a vein graft-associated benefit more strongly than a definitive additive or synergistic benefit of PRF combined with the vein graft.

### qRT-PCR analysis of IL-6 and TGF-β1 relative mRNA expression

**Fig. 10:**
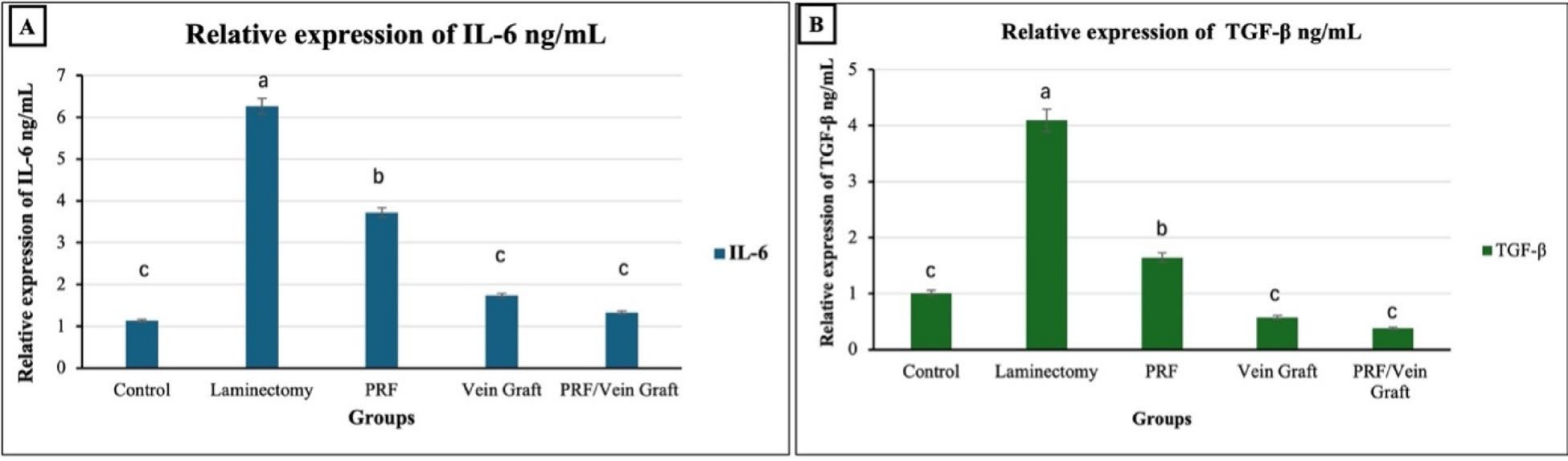
Relative mRNA expression of IL-6 and TGF-β1 in epidural scar tissue, normalized to GAPDH and expressed as fold change relative to the control group. Different superscript letters indicate statistically significant differences among groups based on Tukey’s adjusted post-hoc comparisons at *P* ≤ 0.05. Data are from the histology/molecular subset (n = 10/group).

qRT-PCR analysis was performed using scar tissue collected from the predefined histology/molecular subset (n = 10/group). One-way ANOVA showed significant overall group differences in both IL-6 expression (F (4,45) = 17.87, *P* < 0.0001, η^2^ = 0.61) and TGF-β1 expression (F(4,45) = 97.55, *P* < 0.0001, η^2^ = 0.90; Table 2; Fig. 10A and B). The relative mRNA expression of IL-6 was highest in the laminectomy group (6.264 ± 0.781; 95% CI 4.497–8.031), whereas all treatment groups showed lower expression levels, including PRF (3.719 ± 0.266; 95% CI 3.117–4.320), vein graft (1.734 ± 0.569; 95% CI 0.447–3.020), and PRF/vein graft (1.329 ± 0.436; 95% CI 0.343–2.315). A similar pattern was observed for the relative mRNA expression of TGF-β1, which was highest in the laminectomy group (4.093 ± 0.227; 95% CI 3.580–4.606) and was reduced in the PRF group (1.644 ± 0.151; 95% CI 1.302–1.987), vein graft group (0.576 ± 0.056; 95% CI 0.449–0.702), and PRF/vein graft group (0.381 ± 0.060; 95% CI 0.245–0.517). The magnitude and direction of these reductions support the anti-inflammatory and anti-fibrotic effect of the treatments, with the combined PRF/vein graft group showing the lowest expression levels among the treated groups.

**Table 2:** mRNA expression of IL-6 and TGF-β1 in epidural scar tissue, normalized to GAPDH and expressed as fold change relative to the control group.

Groups	IL-6	TGF-β1
Control	1.1320 ± 0.36061^c^	1.0088 ± 0.18827^c^
Laminectomy	6.2642 ± 0.78088^a^	4.0929 ± 0.22657^a^
PRF	3.7188 ± 0.26589^b^	1.6444 ± 0.15131^b^
Vein Graft	1.7337 ± 0.56878^c^	0.5755 ± 0.05576^c^
PRF/Vein Graft	1.3290 ± 0.43578^c^	0.3812 ± 0.06013^c^

Data are presented as mean ± SE of relative mRNA expression calculated using the 2^-ΔΔCt method after normalization to GAPDH. Different superscript letters within the same column indicate statistically significant differences between groups based on one-way ANOVA followed by Tukey’s adjusted post-hoc test at *P* ≤ 0.05.

## Discussion

Alternative treatment approaches for relieving post-laminectomy epidural fibrosis remain a subject of investigation. The present experiment investigated whether an autologous PRF clot, a femoral vein graft, or their combination could reduce local histological, morphometric, inflammatory, and molecular features of epidural fibrosis after rat laminectomy. The findings support a local anti-fibrotic effect within this short-term experimental model, particularly in the vein graft-containing groups. However, the results do not establish that adding PRF to the vein graft provides a consistent additive or synergistic benefit over the vein graft alone. They also do not establish functional benefit, analgesic efficacy, long-term durability, or readiness for clinical use.

Epidural fibrosis formation occurs when the normal epidural fat is replaced with fibrotic tissue after surgery. This fibrotic tissue binds the dura mater and spinal nerve roots to adjacent structures distally and proximally. This ultimately leads to poor results in spinal cord surgeries^34^. Epidural scar tissue may contribute to FBSS by compressing the dura mater and placing tension on spinal nerve roots. However, FBSS is a multifactorial condition, and other causes such as recurrent disc herniation, spinal instability, stenosis, or incorrect surgical level may also be involved. Traditional treatment and surgical scar resection are the current therapeutic regimens available. Unfortunately, they are not satisfactory^35^.

Different techniques have been investigated to reduce epidural fibrosis and adhesions. These methods include the use of physical barriers composed of biomaterials and topical or systemic medication therapy^36–38^. However, a limited number of drugs and biomaterials have been included in clinical trials. Currently, there are very few options available for preventing epidural fibrosis and epidural adhesions following a laminectomy. Epidural fibrosis and epidural adhesions continue to pose a significant treatment challenge, underscoring the urgent need for the development of effective anti-fibrosis medications. Therefore, the present study aimed to reduce EF in a rat model using a femoral vein graft and PRF-based treatments.

Fibroblast trans-differentiation, proliferation, and excessive extracellular matrix protein deposition are pathogenic processes associated with epidural fibrosis and adhesions. TGF-β1 is a potent fibrotic cytokine that plays a crucial role in promoting the proliferation of fibroblasts^39^. This proliferation is intended to repair the locally defective vertebral region where a laminectomy is performed^3^.

In our study, we used male Sprague Dawley rats because endogenous estrogen may impact EF formation following lumbar laminectomy in rats^40^. Rats have become increasingly valuable in research due to their ease of maintenance and acquisition, low cost of care, and high disease resistance^41^.

PRF resulted in a reduction of both inflammation and the formation of epidural scar tissue surrounding the spinal cord, in comparison to the group that underwent laminectomy. The observed effects can be ascribed to angiogenesis, enhanced osteoblast proliferation, reduced bleeding in epidural space, and tissue healing facilitated by cytokines^20,42^. The growth factor released from platelets indicates that fibrin-containing gels alleviate EF within the first two weeks following a laminectomy. Moreover, the growth factor found in platelet concentrate decreases fibrotic connective tissue by promoting reperfusion of damaged muscle tissue^43^. Furthermore, IL6 and TGFβ-1 were markedly eliminated in PRF-treated rats than in the laminectomy group. This was done to validate the histopathological scoring related to the aforementioned advantages of PRF.

The histological epidural fibrosis endpoint, supported by the secondary macroscopic, morphometric, and molecular findings, suggests that PRF contributes to soft tissue regeneration and wound healing, although the strongest anti-fibrotic effect was observed in the vein graft-containing groups. In addition, PRF is a safe, reliable, and cost-effective option for expediting wound healing and enhancing the efficiency of tissue repair following damage or injury^44^. Based on Rydell’s classification, among rats treated with PRF after laminectomy, 20% showed epidural scar adhesion grade 0, 50% grade 1, 20% grade 2, and 10% grade 3. This outcome demonstrates that PRF effectively reduces epidural scar adhesion in comparison to the laminectomy group. This result confirms the previous finding^20^ that PRF eliminates epidural adhesion by alleviating fibroblast proliferation and inflammatory cell infiltration.

The mechanism by which the femoral vein graft reduced epidural fibrosis in the present model remains uncertain. In this study, the vein graft was placed over the exposed dura after laminectomy; therefore, its biological context differs from that of vein wrapping or vein conduits used in peripheral nerve regeneration^19,45–48^. The reduced fibrosis and adhesion observed in the vein graft-containing groups may be partly related to a local barrier effect that physically separates the dura from overlying postoperative scar tissue, which is consistent with the general concept of barrier-based strategies for limiting epidural adhesion formation^38,45^. Additional biological effects, such as modulation of local inflammation, fibroblast infiltration, extracellular matrix deposition, or vascularized wound healing, are possible because these processes are involved in epidural fibrosis formation^17,18,39^, but they were not directly tested in the present study. Therefore, references from peripheral nerve regeneration should be interpreted only as supportive background for the biocompatibility of autogenous vein tissue, not as direct mechanistic evidence for epidural fibrosis prevention after laminectomy^19,45–48^. Future studies should directly assess the mechanism using markers of fibroblast activation, myofibroblast differentiation, collagen deposition, macrophage polarization, angiogenesis, and graft integration/remodeling at the laminectomy site. The present study showed that the inflammation, scar density, and dura matter thickness were the lowest grades in animals treated with vein grafts. These results were consistent with previous reports^15,49–51^. They suggested that venous grafts can facilitate graft invasion by increasing Schwann cell motility, decreasing fibroblast infiltration, preventing uncontrolled fiber outgrowth, and accelerating neovascularization and growth factor secretion.

On the other hand, the tunica adventitia of the vein is responsible for nerve regeneration by providing a neurotrophic medium, as it is primarily high in collagen content. Additionally, the tunica intima contains a laminin-rich basal layer, which is a complex glycoprotein and is the major non-collagenous component, as reported by^45^.

The qRT-PCR findings, calculated using the 2^-ΔΔCt method after normalization to GAPDH^34^, should be interpreted as relative mRNA expression levels in the collected epidural scar tissue. Therefore, the reduced IL-6 and TGF-β1 expression observed in the treated groups may reflect both lower inflammatory/fibrotic activity and the reduced overall amount of scar tissue at the laminectomy site.

Importantly, the interpretation of the present findings was not based on statistical significance alone. The treatment effects were also supported by the magnitude and direction of change across macroscopic, histological, morphometric, and molecular endpoints. For example, severe grade 3 epidural adhesion predominated in the laminectomy group, whereas the vein graft and PRF/vein graft groups showed a marked shift toward grade 0 or grade 1 adhesion. Similarly, dura mater thickness and IL-6/TGF-β1 expression showed large reductions in the treated groups compared with the laminectomy group. Therefore, the biological relevance of the findings is supported by consistent reductions in severity and measurable effect magnitude, not by *P* values alone.

Although the present findings demonstrate that vein graft-containing treatments can attenuate epidural fibrosis and adhesion formation in a rat laminectomy model, the added value of combining PRF with the vein graft remains uncertain because the combined group was often comparable to the vein graft-alone group. Clinical translation of these findings to human laminectomy patients should therefore be approached with caution. From a clinical perspective, harvesting an autologous femoral vein graft solely to prevent epidural fibrosis after routine lumbar surgery may be impractical without clear evidence that the benefit outweighs the added surgical burden. Donor-site morbidity, increased operative time, graft handling, and the creation of a second operative site must be considered before such an approach can be translated to patients. Previous reports on autologous vein harvesting have described donor-site concerns, including postoperative leg swelling, wound complications, pain or paresthesia, and possible chronic edema, particularly when more extensive vein harvesting is performed^52,53^. Therefore, the present findings should be viewed as preliminary proof-of-concept evidence supporting further investigation of vein graft-based barrier strategies, not as a recommendation for routine clinical use of autologous femoral vein grafts in lumbar surgery. Future studies should also evaluate clinically feasible alternatives, including optimized graft preparation, less morbid autologous sources, or biomaterial-based substitutes that could reproduce the barrier effect without requiring additional vein harvesting.

Epidural fibrosis in humans is clinically heterogeneous and influenced by multiple patient- and procedure-related factors that are not fully reproduced in young, otherwise healthy rodents. Differences in epidural anatomy, wound-healing kinetics, immune response, comorbid conditions, and variability in surgical techniques may limit direct extrapolation. In addition, the present study evaluated outcomes at a single postoperative time point and did not include long-term functional neurological assessment, behavioral pain testing, dose–response evaluation, or durability analysis. Therefore, the present findings should be interpreted as preliminary experimental evidence rather than direct clinical evidence.

Another important limitation is the absence of a sham-operated surgical control. The normal, untouched control group provided baseline histological and molecular values, but it cannot separate the effects of anesthesia, surgical exposure, paraspinal muscle dissection, bleeding, and wound healing from laminectomy-related fibrosis. Therefore, comparisons with the normal control group should be interpreted only as baseline reference comparisons. The laminectomy-only group remains the appropriate surgical comparator for assessing the relative anti-fibrotic effects of PRF, vein graft, and combined PRF/vein graft treatment after laminectomy. Future studies should include a sham-operated group to better distinguish nonspecific surgical wound-healing effects from fibrosis specifically induced by laminectomy.

Despite these limitations, the observed reductions in histological fibrosis, adhesion severity, dura mater thickness, and IL-6/TGF-β1 expression suggest that vein graft-containing strategies may warrant further preclinical investigation. However, these findings do not prove that PRF/vein graft treatment is clinically effective for preventing symptomatic epidural fibrosis after laminectomy, nor do they establish a consistent superiority of the combined treatment over vein graft alone. This distinction is important because many anti-fibrotic strategies for post-laminectomy epidural fibrosis have shown promising experimental results but limited or uncertain clinical translation^10–13^. Therefore, future studies should include sham-operated controls, standardized comparisons of PRF dose and handling, assessment of graft orientation and fixation, longer follow-up, functional neurological outcomes, pain-related behavior, and large-animal models before clinical application is considered.

## Conclusion

In this rat laminectomy model, local vein graft-containing treatments reduced epidural fibrosis-related histological scores, dura mater thickness, and the relative mRNA expression levels of IL-6 and TGF-β1 at 30 days. The combined PRF/vein graft treatment showed favorable short-term local anti-inflammatory and anti-fibrotic effects and was generally more effective than PRF alone; however, it was not consistently superior to vein graft alone across all endpoints. Thus, the results support the potential value of vein graft-based local barrier strategies, while the specific additive benefit of PRF requires further investigation. Because the study did not include functional neurological outcomes, pain behavior assessment, long-term durability testing, PRF dose/handling comparisons, assessment of donor-site morbidity, operative feasibility, or a sham-operated surgical control, these findings should be considered preliminary preclinical evidence rather than support for clinical application in human laminectomy patients.

## Data Availability

All data supporting the findings of this study are available within the paper.

## Abbreviations

PRF: Platelet-rich fibrin
FBSS: Failed back surgery syndrome
EF: Epidural fibrosis
TGF-β1: Transforming growth factor-1beta
IL-6: Interleukin-6
EAS: Epidural adhesion score
